# Combination of bone marrow mesenchymal stem cells and cartilage fragments contribute to enhanced repair of osteochondral defects

**DOI:** 10.6026/97320630013196

**Published:** 2017-06-30

**Authors:** Mohammed Abbas

**Affiliations:** 1 Department of Orthopaedic Surgery, Faculty of Medicine, King Abdulaziz University, Jeddah, Saudi Arabia; 2 Sheikh Salem Bin Mahfouz Scientific Chair for Treatment of Osteoarthritis by Stem Cells, King Abdulaziz University, Jeddah, Saudi Arabia

**Keywords:** Osteoarthritis, mesenchymal stem cells, tissue engineering, cartilage repair, osteochondral explants

## Abstract

Cartilage tissue engineering using stem cells and biomaterials is considered a promising approach despite poor outcomes. We hypothesise that articular cartilage fragments provides native environmental cues to enhance stem cell differentiation. As such we evaluated the chondrogenic differentiation and repair of critical size defect in a human explant osteochondral model (OD) using bone marrow derived mesenchymal stem cells (BM-MSCs) and homogenised cartilage. BM-MSCs were established from the bone-marrow plugs of patients undergoing total knee arthroplasty and characterized. Osteochondral tissue was trimmed and a central drill defect (∼2mm) was made. Chondrogenic repair was evaluated by filling the OD defect area with either BM-MSCs (Group II), homogenized cartilage (Group III) or a combination of both BM-MSCs and homogenized cartilage (Group IV). OD with no added cell or tissue served as control (Group I). Samples were maintained in chondrogenic differentiation medium for 28 days. Microscopic images showed maximal OD closure in Group IV. Partial OD closure was observed in Group II and to a lesser extent in Group III. Haematoxylin-eosin staining revealed immature cartilaginous matrix in Group II and more mature matrix in Group IV. Sircol™ Assay showed increased collagen deposition in both Group II and Group IV. Immunostaining for both groups revealed positive staining for type II collagen. Combining BM-MSCs and homogenised cartilage demonstrated enhanced cartilage formation and defect filling in a human ex-vivo osteochondral model.

## Background

Osteoarthritis (OA) is a progressive degenerative disease of the joint characterized by gradual degradation of the cartilaginous extracellular matrix (ECM) and sclerosis of bone. The ECM of cartilage is highly specialized structure that is mainly composed of type II collagen that provides tensile strength and proteoglycans that provide compressive stiffness [1]. The imbalance in the turnover of proteoglycans and type II collagen network leads to loss of cartilage integrity and hence its function [2]. Cartilage has a limited ability to repair itself and restore the articular surface.

Mesenchymal stem cells (MSCs) are considered a promising candidate for cartilage regeneration, due to their self-renewal capacity and potential to differentiate into chondrocytes, as well as other cell types [3]. Moreover, MSCs secrete abundant trophic factors that that support their tissue maintenance and regenerative functions [4]. Recent evidence points that native ECM plays a crucial role in dictating cell differentiation towards the desired lineage by acting in concert with the soluble molecules [5]. In cartilage repair, ECM may be responsible for guiding the chondrogenic differentiation of MSCs [6-8]. This has guided many groups to design synthetic and natural constructs that mimic individual components rather than whole native articular cartilage ECM to promote cartilage regeneration [5]. Such individual component (e.g. collagen or hyaluronic acid) or combination thereof provides limited substrate for the necessary cellular cues to aid differentiation, maturation, and remodeling of repair tissue [9]. Moreover, studies that explored the potential of whole ECM infused with MSCs were limited to assessing chondrogenesis rather true defect repair [6-8]. In this short report, we evaluated the chondrogenic differentiation and repair of critical size defect in a human explant osteochondral model using bone marrow derived mesenchymal stem cells (BM-MSCs) and homogenized cartilage.

## Methodology

The present study was approved by the Ethical Committee for Scientific Research of King Abdulaziz University, Jeddah, Saudi Arabia [No. 113-157]. Informed consent was obtained from all patients before bone marrow aspiration, and collection of osteochondral plugs and cartilage from undamaged areas from patients undergoing total knee arthroplasty.

### Derivation of BM-MSCs

BM-MSCs were isolated from six patients according to earlier established protocols [10]. Briefly, bone marrow aspirates collected in heparinzed tubes (Becton Dickinson, New Jersey, USA) and directly plated (∼2ml) into T175cm2 flasks (Greiner Bioone). The cells were cultured using Dulbecco's modified Eagles medium (DMEM; Sigma, Missouri, USA) supplemented with 10% (v/v) gamma irradiated foetal bovine serum (Sigma, Missouri, USA), 2mM GlutaMax (Invitrogen, Life Technologies, California, USA), and antibiotic solution [penicillin (100IU/mL; streptomycin (100μg/mL); Sigma, Missouri, USA]. The monolayer of adherent cells was cultured and propagated under standard culture conditions of 37°C and 5% CO2 in atmospheric air.

### Cell morphology and proliferation

BM-MSCs were plated at a seeding density of 2x104 cells per well in a 24 well plate and cultured under standard culture conditions for 72 h. Cell morphology was imaged using a phase contrast microscope and cell proliferation was determined at 24 h, 48 h and 72 h using MTT reagent (3-(4,5-dimethylthiazolyl-2)-2,5- diphenyltetrazolium bromide; Sigma, MO) and absorbance was obtained at 570 nm (reference 630 nm) using a spectrophotometer (SpectraMax® i3x, Molecular Devices, Sunnyvale, CA).

### Surface marker analysis

The derived BM-MSCs were characterized for their expression of MSC related surface antigens using fluorescence-activated cell sorting (FACS) analysis. Briefly, BM-MSCs was aliquoted and stained with MSC isotype, positive (CD73, CD90, CD105, CD44, CD29) and negative (CD34, CD45) antibodies (Miltenyi Biotec) at 1:10 dilution for 15 min at 4°C. The cells were then washed once with 3% (v/v), centrifuged (500 g for 5 min) and the pellet resuspended in 3% (v/v) FBS and analysed using FACS Aria III (BD Biosciences).

### BM-MSCs differentiation into adipocytes, osteoblasts and chondrocytes

Expanded BM-MSCs (2 x 104 cells/well) were seeded into a 24 well plate and allowed to reach confluence. They were then cultured using StemPro° adipocyte, osteoblast and chondrocyte differentiation media for up to 21 days with fresh media changes every 3 - 4 days. Following differentiation, the cells were fixed in 4% formaldehyde solution for 30 minutes and rinsed with PBS twice. The cells were then stained with oil Red O (adipocytes), Alizarin red (osteoblasts) or Alcian blue (chondrocytes) (Sigma), washed and analysed for positive staining using light microscopy.

### Chondrogenic repair of an ex vivo osteochondral defect

Osteochondral bone samples that were obtained from patients undergoing total knee arthroplasty were trimmed to 1cm (w) x 1cm (b) x 1cm (h) and a central drill defect (∼2 mm) was made. Chondrogenic repair was conducted by seeding the osteochondral defect (OD) with BM-MSCs (1x106cells) pellet (Group-II), homogenized cartilage pellet (Group-III) or a combination of both BM-MSCs (0.5x106cells) and homogenized cartilage pellet (Group-IV). OD with no added cell or tissue served as control (Group-I). Fresh undamaged articular cartilage from the femoral condyles were finely homogenized and pelleted by centrifugation. The pellet was re-suspended in 5 ml of PBS and aliquots of 250 μl were pelleted and used either separately (Group-III) or together with MSCs (Group-IV) respectively. Similar sized cell pellets were used for all experimental conditions. Samples from all four groups were maintained in standard BM-MSC chondrogenic medium up to 28 days. Tissue formation and repair of the central drill defect was analysed by phase contrast imaging, histology, immuno-histo-chemistry and biochemical analysis.

### Histology and immuno-histo-chemistry

Histological analysis was performed following the differentiation period of 28 days. Briefly, the ex-vivo samples were fixed in 10% (v/v) neutral buffered formalin for 24 h at 4°C, demineralized using an aqueous mixture of 5% (v/v) formic acid and 5% (v/v) formalin. Demineralized samples were post-fixed in 70% (v/v) ethanol before being embedded in paraffin. Tissue were sectioned at 10 μm thickness and stained with Toluidine blue for histological analysis. For type II collagen immunostaining, the tissue sections were deparaffinized in xylene, washed in graded series of ethanol (100%, 95%, 70%) and rehydrated. Antigen retrieval was done by heating the tissue in sodium citrate buffer (pH 6.0) using microwave for 15 min and the tissue was left to cool for at least 30 min. The tissue was blocked with peroxidase before incubating with mouse monoclonal anti-collagen II (Abcam) at 1:100 dilution at 4°C overnight. The sections were washed and incubated with respective secondary goat antimouse antibodies for 30 min (Dylight 488 and Dylight 405, Biolegend). The tissue was finally counterstained with DAPI and analysed using fluorescent microscope (EVOS).

### Collagen (SircolTM) assay

Secreted collagen levels from all groups were evaluated using Sircol (collagen assay) kit (Bioclor) according to the manufacturer׳s instructions. Briefly, 1 ml of the Sircol reagent was mixed with 100 μl of the standards and samples (1:20, diluted in distilled water) to form collagen-dye complex. They were then centrifuged at 12000 rpm for 10 min and the supernatant removed. Unbound dye was removed by layering 750 μl of ice-cold acid-salt wash reagent (Kit content) followed by centrifugation (12000 rpm for 10 min) and removal of the supernatant. Alkali reagent (250 μl) was added to dissolve the bound dye and absorbance at 555 nm was spectrophotometrically measured using a micro-plate ELISA reader (μQuant-BioTek) to determine collagen concentration.

### Statistical analysis

Comparisons between treatment and control for cell proliferation and collagen assays were analysed using One-Way ANOVA test with the Statistical Package for Social Sciences (SPSS 21). The results were expressed as mean ± standard error of the mean (SEM) from three different replicates for individual assays and a value of p<0.05 was considered to be statistically significant.

## Results

### BM-MSC morphology and growth characteristics

**Figure 1:**
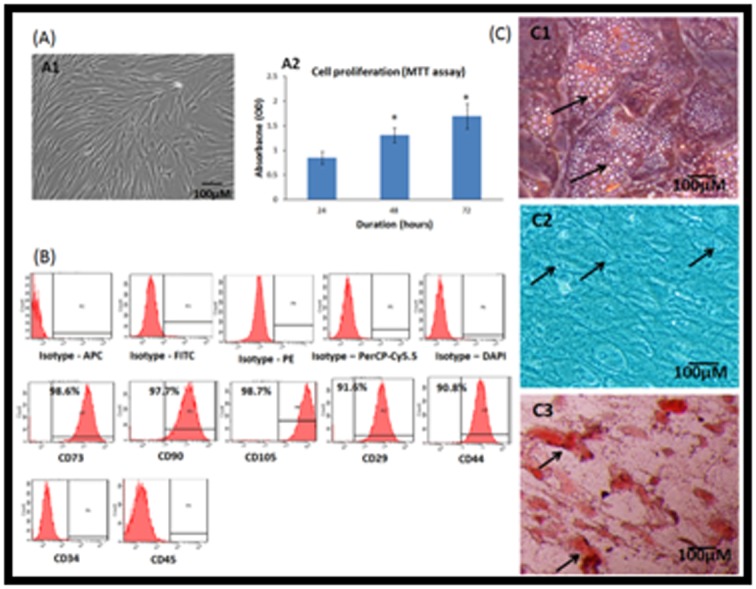
A - Phase contrast image of bone marrow mesenchymal stem cells (BM-MSCs) showing the characteristic fibroblastic morphology in monolayer culture (A1); Cell proliferation of the BM-MSCs by MTT assay at 24 h, 48 h and 72 h showing increase in cell numbers with increase in time (A2). Values are expressed as mean ± SEM of three independent samples and asterisk (*) indicates statistical significance (P<0.05); B - Representative Fluorescent activated cell-sorting (FACS) analysis showing the CD marker expression pattern in human bone marrow mesenchymal stem cells (hBM-MSCs). Top panel: Respective isotype controls; Middle panel: MSC positive CD markers; Bottom panel: MSC Negative CD markers; C - Histological images of the human bone marrow mesenchymal stem cells (hBM-MSCs) differentiated into (C1) adipocytes, (C2) chondrocytes and (C2) osteoblasts and stained with oil red O, Alcian blue and Alizarin red stains respectively. Arrows indicate fat cell vacuolations (A); chondrocyte like cells (B) and areas of calcium mineralization (C). Magnification 10X.

BM-MSCs adhered to the culture surface as multiple patches as early as day 4, while the non-adherent cells were gradually lost with changing of media. The cell numbers continued to increase reaching up to 70% - 80% confluence by the second week. The BM-MSCs showed characteristic spindle shape in culture (Figure 1 A1). The initial number of cells in primary monolayer cultures varied from 0.9±0.2-to 1.2±0.4 x 106 cells/2mL bone marrow aspirate. Cell numbers showed marked expansion following subcultures showing a uniform monolayer of cells and they were used in subsequent experiments. BM-MSCs demonstrated an increase in cell numbers from 24 h - 72 h. There was a statistically significant mean increase by 58.33% and 108.33% at 48 h and 72 h respectively (Figure 1 A2).

### Surface Maker analysis

Primary cultures from bone marrow aspirates analysed for surface marker expression demonstrated high percentages of MSC related positive markers, namely CD73 (98.6%), CD90 (97.7%), CD105 (98.7%), CD44 (90.8%) and CD29 (91.6%) compared to their respective isotype matched controls. The cells were negative for CD34 and CD45, the haematopoietic stem cells related surface markers (Figure 1B).

### Differentiation into adipocytes, chondrocytes and osteoblasts

Primary cultures of BM-MSCs showed differentiation into adipocytes, chondrocytes and osteoblasts upon culture in respective differentiation medium (StemPro°; Figure 1C). Lipid vacuolations were observed following culture in adipocytic differentiation medium for three weeks. These cells stained positive with oil red O (Figure 1 C1). Chondrocyte like cells were observed following culture of BM-MSCs in chondrogenic differentiation medium for up to 21 days. The differentiated cells showed positive staining with Alcian blue (Figure 1 C2). Cell aggregation and mineralization were observed following osteogenic differentiation of BM-MSCs and these showed positive staining with Alizarin red (Figure 1 C3).

### Ex-vivo cartilage repair

**Figure 2:**
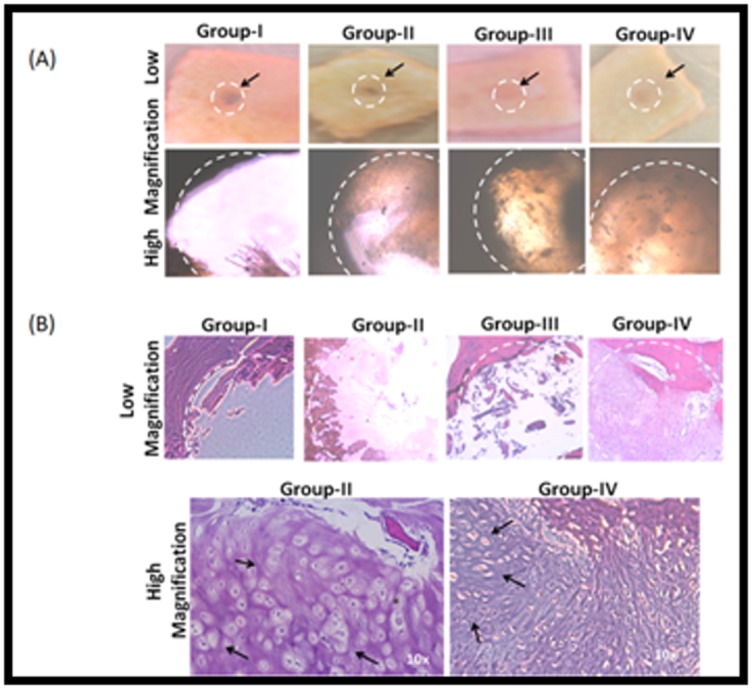
A - Gross images of the explant culture after 14 days (low and high magnification) showing the osetochondral bone with central drill defect (circular dotted lines) in different groups. Group-I (control); Group-II (BM-MSCs); Group-III (cartilage) and Group- IV (BM-MSCs + cartilage). In the control (Group I) the defect area remains empty, whereas the other groups (Group-II, Group-III and Group-IV) shows varying degrees of repair of the defect area with respective cell types; B - Haematoxylin and eosin (H &E) staining of demineralized tissue sections of the paraffin embedded bone tissue following 28 days of ex-vivo at lower magnification; C - H & E images of Group-IV (BM-MSCs + cartilage homogenate) at higher magnification. Arrows indicate chondrocyte.

Phase contrast (Figure 2A) and H&E staining (Figure 2B) images of the 28 days cultured explants showed varying degrees of OD closure. The areas within the dotted lines indicate the defect area. Images for Group I show that the defect area appears empty compared to other groups. Group II demonstrated partial filling of the OD closure and Group III demonstrated very little presence of filling matrix around the edges of the defect while Group IV filled approximately 90% of the defect area. This was supported by histological staining with H and E (Figure 2B). The staining demonstrated that Group I was devoid of cells in the defect area while the rest of the groups showed varied presence of cells/tissue filling up the defect. Higher magnification of the repair tissue revealed the presence of BM-MSC-derived chondrocytes embedded in lacunae in Group-IV (Figure 2C). Group II that was filled with BM-MSC pellets alone and Group- III that was seeded with homogenised cartilage alone both did not contribute to any significant defect filling (Figure 2B).

### Characterization of repair tissue

**Figure 3:**
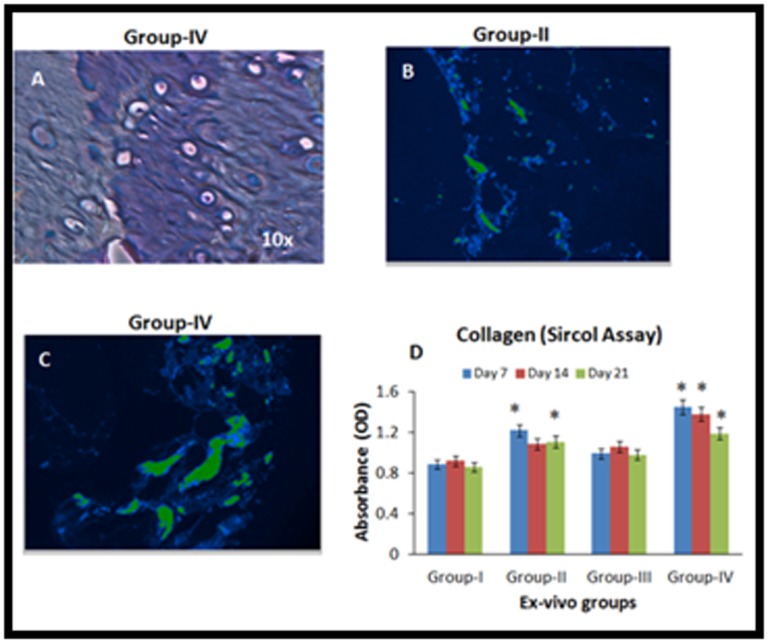
A - Toluidine blue staining of demineralized tissue sections of the paraffin embedded bone tissue following 28 days of ex-vivo culture showed positive staining indicative of collagen content; B, C - Immuno-histo-chemistry images showing positive staining for human collagen II in Group II (BM-MSCs) and Group-IV (BM-MSCs + cartilage homogenate); D - Sircol (collagen) assay showing the secreted collagen levels at days 7, 14 and 21 from various treatment groups. The values are shown as mean ± SEM from three independent samples. Statistical analysis was conducted using one-way ANOVA test. Asterisk (*) indicates statistical significance (P<0.05).

Toluidine blue staining of Group-IV demonstrated positive staining of proteoglycans indicative of chondrogenic differentiation (Figure 3A). Immunohisto- chemistry for human type II collagen of tissue sections obtained from Group-II showed positive staining (Figure 3B). That staining appears more intense in Group-IV (Figure 3C). Secreted collagen measured at days 7, 14 and 21 using Sircol™ assay showed increased release in treated groups (Group-II, Group-III and Group-IV) compared to control (Group-I) indicating new matrix synthesis and turnover (Figure 3D). There was statistically significant increase in turnover in groups where BM-MSCs were present (i.e. Groups II and IV). The increase in these groups decreased over time in line with decreased synthesis and repair tissue maturation (Figure 3D).

## Discussions

We have demonstrated in this study for the first time that cartilage repair in an ex vivo human osteochondral defect model can be achieved using a mixture of BM-MSCs and homogenised native cartilage. The repair tissue of mixed BM-MSCs and homogenised cartilage appears to be stable, integrating with surrounding host tissue and capable of turnover. This finding suggests it will be possible to develop a method for robust cartilage repair using native tissue and BM-MSCs.

The BM-MSCs derived and used in this study satisfied the minimal criteria for MSCs as stated in the position paper [10]. The explant tissue used in our study was osteochondral therefore it is a relevant model for natural knee anatomy accounting for cartilage and bone contribution. The chondrogeneic differentiation of BM-MSCs depends on cues from the local micro-environmental cues, including stem cell factors, growth factors such as bone morphogenic proteins, transforming growth factor beta, presence of native osteoblasts as well as chondrocytes [11, 12]. The enhanced chondrogenic differentiation observed in our study in Group-II (BM-MSCs) and in Group-IV (BM-MSCs + cartilage fragments) (Figure 2) and the positive staining for collagen type II antibody (Figure 3) support this finding.

The osteochondral length is comprised of highly organized structures: the elongated chondrocytes and collagen fibrils that run parallel in the upper zone, the intermediate zone of round chondrocytes and less of collagen fibrils, and the deep zone where chondrocytes and collagen fibrils are arranged in vertical columns perpendicular to the articular surface [3, 13]. Deep zone contributes to the highest secretion of proteoglycans and together with collagen, are essential for the maintenance of structure and function of the cartilage tissue. Our study identified that the secretory levels of collagen was higher in Group-II (BM-MSCs) and in Group-IV (BM-MSCs + cartilage homogenate) compared to other groups (Figure 3D). The presence of BM-MSCs appears to be critical for having a competent repair tissue that can turnover and interact with the chondral and subchondral layers [14, 15]. Similarly, native cartilage homogenate appears to be critical for a robust tissue repair using BM-MSCs. Previous work demonstrated the positive impact of cartilage fragments on MSC chondrogenic differentiation in pellets without proving the viability of this approach in a bona fide chondral or osteochondral defect or characterizing this repair tissue in situ [8].

A recent study has highlighted the importance of having a healthy BM-MSC in the subchondral niche to avoid progression of osteoarthritis and suggested that manipulation of subchondral MSCs could be a treatment option for osteoarthritis [16]. An earlier in vitro study has reported that bone tissue plugs can preserve chondrocyte survival [13]. In our study, the use of healthy subchondral bone tissue could have established cross talk and signaling between the stem cells, cartilage fragments and surrounding bone tissue leading to better chondrogeneic differentiation.

## Conclusion

The results from the present study showed that the combination of BM-MSCs and cartilage fragments provides better cartilage repair. This is in part due to the presence of micro-environmental cues provided by the native cartilage tissue, which facilitated effective interaction between the bone and cartilage in this human ex-vivo osteochondral defect model. Further analysis of sub-fragments could help design more effective methods to understand the signaling mechanisms involved in enhanced cartilage repair and pave way for the delivery of therapeutic chondrogenic MSCs.
